# Maxillary protraction after surgically assisted maxillary expansion

**DOI:** 10.1590/S1678-77572010000300020

**Published:** 2010

**Authors:** Laurindo Zanco FURQUIM, Guilherme JANSON, Bruno D’Aurea FURQUIM, Liogi IWAKI FILHO, José Fernando Castanha HENRIQUES, Geovane Miranda FERREIRA

**Affiliations:** 1 DDS, PhD, Private Practice.; 2 DDS, MSc, PhD, MRCDC (Member of the Royal College of Dentists of Canada), Professor and Head, Department of Pediatric Dentistry, Orthodontics and Community Health, Bauru School of Dentistry, University of São Paulo, Bauru, SP, Brazil.; 3 DDS, Orthodontic Graduate Student, Department of Pediatric Dentistry, Orthodontics and Community Health, Bauru School of Dentistry, University of São Paulo, Bauru, SP, Brazil.; 4 DDS, MSc, PhD, Private Practice, Maringá, PR, Brazil.; 5 DDS, MSc, PhD Professor, Department of Pediatric Dentistry, Orthodontics and Community Health, Bauru School of Dentistry, University of São Paulo, Bauru, SP, Brazil.; 6 DDS, Private Practice, Maringá, PR, Brazil.

**Keywords:** Extraoral traction appliances, Class III malocclusion, Adult

## Abstract

This case report describes the orthodontic treatment of a 32-year-old woman with a Class III malocclusion, whose chief compliant was her dentofacial esthetics. The pretreatment lateral cephalometric tracings showed the presence of a Class III dentoskeletal malocclusion with components of maxillary deficiency. After discussion with the patient, the treatment option included surgically assisted rapid maxillary expansion (SARME) followed by orthopedic protraction (Sky Hook) and Class III elastics. Patient compliance was excellent and satisfactory dentofacial esthetics was achieved after treatment completion.

## INTRODUCTION

Potpeschnigg^16^ (1875) first described the protraction facemask in 1875 and Delaire, et al.^4^ (1976) revived the interest in maxillary protraction 100 years later. Protraction facemask in conjunction with a maxillary expansion appliance has been used to correct malocclusions associated with maxillary deficiency and/or mandibular prognathism, disarticulating maxillary sutures and allowing an efficient forward protraction of the maxilla^11-14,19^.

More recently, Daher, et al.^3^ (2007) used the facemask therapy in a non-surgical treatment of an adult patient, to provide dentoalveolar compensation. The use of extraoral traction with a Delaire-type facemask in combination with a maxillary corticotomy following the design of a Le Fort I osteotomy has been proposed in adolescents^15^ and adults^2^. Resistance to maxillary protraction by the craniofacial skeletal architecture could be reduced by using osteotomic cuts which allow true progress in orthopedic advancement with almost exclusively skeletal effects and a reduction of the risk of relapse.

This paper presents the case of an adult patient with Class III malocclusion who was reluctant to undergo orthognatic surgery, as was treated with surgically assisted rapid maxillary expansion (SARME) followed by maxillary orthopedic protraction. The SARMe was undertaken in a private dental practice under local anesthesia.

## CASE REPORT

**Figure 1:**
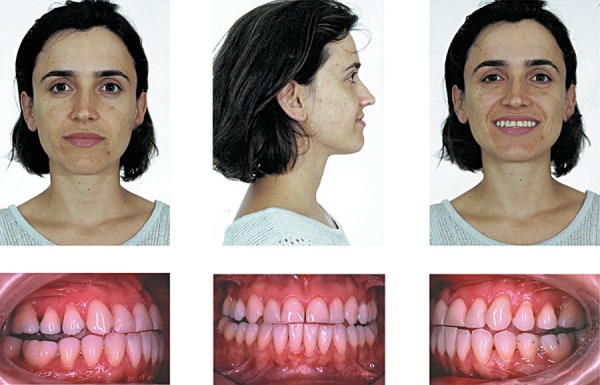
Pretreatment facial and intraoral photographs (patient signed informed consent authorizing the publication of these pictures)

**Figure 2:**
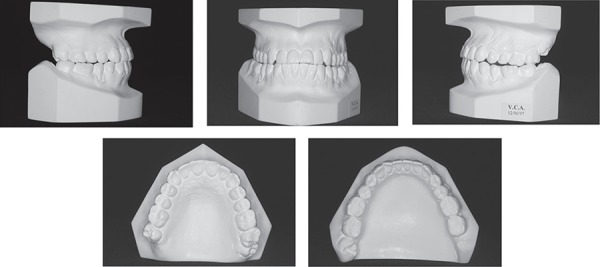
Pretreatment study models

A 32-year-old woman presented for orthodontic treatment at Dr. Laurindo Zanco Furquim's private practice. Her chief complaint was her facial esthetics. Clinical examination confirmed a concave profile, retruded upper lip and procumbent lower lip. The patient had a complete dentition up to the second molars, with a bilateral Class III dental relationship. Intraoral and the dental cast examinations revealed an absolute transverse deficiency of the maxilla. The compensatory tipping of the maxillary and mandibular incisors resulted in normal incisor relationship despite the deficient sagittal jaw relationship (Figures 1 and 2). The pretreatment lateral cephalometric tracings showed the presence of a Class III dentoskeletal malocclusion with components of maxillary deficiency (Table 1).

**Table 1:** Pretreatment, posttreatment and follow-up cephalometric values

**Measurement**	**Pretreatment**	**Posttreatment**	**Follow up**
			
Maxillary component			
SNA	77.2°	78.7°	78.7
A-Nperp	-6.6 mm	-5.1 mm	-5.2 mm
Co-A	79.5 mm	81.1 mm	81 mm
Mandibular component			
SNB	81.8°	81.1°	81.2
P-Nperp	-1.3 mm	1.2 mm	-1 mm
P-NB	2.9 mm	4.3 mm	4.2 mm
Co-Gn	117.1 mm	116.8 mm	116.6 mm
Maxillomandibular component			
ANB	-4.7°	-2.4°	-2.6
Profile convexity			
NA-NPo	-12.1°	-9.2°	-9.5
Vertical component			
FMA (MP-FH)	27.6°	26.9°	28.6
SN-OP	6.7°	8°	8.3
ANS-Me	65.2 mm	64.1 mm	64.4
Maxillary dentoalveolar component			
U1.NA	43.3°	39.1°	37.9
U1-NA	11.9 mm	10.3 mm	10.5
Mandibular dentoalveolar component			
L1.NB	19.6°	25.4°	25.1°
L1-NB	3.5 mm	4.4 mm	5.1 mm
IMPA	84.2°	90.5°	89.2°
Interdental			
Overjet	0 mm	0 mm	0 mm
Overbite	0 mm	3 mm	2 mm
Interincisal angle	120.1°	117.9°	119.5°
Molar relationship	Class III subd. Right	Class I	Class I
Soft tissue			
UL to E-Plane	-8.7 mm	-7.5 mm	-7.2
Mentolabial sulcus	136°	132°	133°
Nasolabial angle	94°	99°	99°

Overall treatment goals consisted of correcting the compensatory tipping of the mandibular incisors and the A-P basal relationship by advancing the maxilla. These changes were expected to greatly improve the patient’s facial esthetics. Limited treatment objectives were to correct the occlusal discrepancies by means of dentoalveolar compensation, which would produce some facial improvement.

Based on the objectives, 3 treatment options were proposed. A compromised treatment by means of dentoalveolar compensation was the first considered option. Secondly, to attain the overall objectives, combined surgical and orthodontic treatment with maxillary expansion and advancement was proposed. However, the risks and treatment expenses would be high. The third option consisted of surgically assisted maxillary expansion followed by orthopedic protraction and A-P discrepancy correction by means of maxillary and mandibular dentoalveolar compensation. Although the risks and costs of this option were lower than the other options, it demanded more time and high patient compliance.

The patient chose the third option because she thought that the possible esthetic improvement with surgery was not worth the high cost and risk. She was reluctant to undergo extensive surgical procedures and was willing to accept a less-thanideal result. Therefore, orthodontic treatment with maxillary expansion followed by orthopedic protraction with Sky Hook appliance was performed to correct the inadequate occlusal relationship and to improve her facial esthetics.

The technique used for maxillary expansion is a variation of that proposed by Bays and Greco^1^ (1992), under local anesthesia. The surgical technique consists of a maxillary lateral wall osteotomy extended posteriorly to the tuber avoiding the pterygomaxillary fissure. The Hyrax appliance was cemented to the first premolars and first molars on each side a few days before surgery. The expander must have an extension to the second premolars and canines, and hooks for the protraction. Five days after surgery, the Hyrax was activated two quarters twice a day (1 mm *per* day) for eleven days. The Sky Hook headgear was used for maxillary protraction according to Haas protocol^5^.

**Figure 4:**
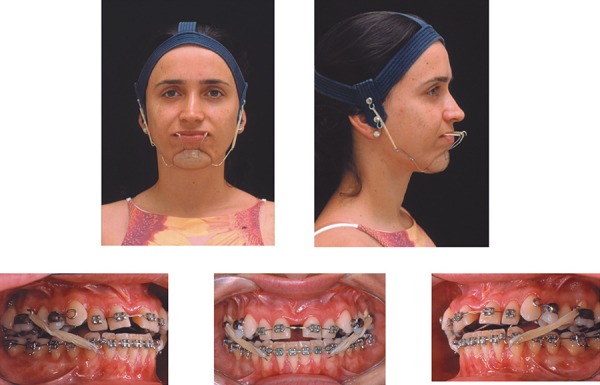
Treatment facial and intraoral photographs (patient signed informed consent authorizing the publication of these pictures)

Straight-wire Capelozza prescription Class III brackets were applied (lingual crown torque on the mandibular anterior teeth of -6°; and mandibular canine slots angulated 0°). Leveling and alignment of the mandibular arch began with rectangular 0.016 X 0.022-inch heat-activated NiTi archwire, simultaneously with maxillary expansion, which allowed the use of Class III elastics, full time, except during meals. The Sky Hook was used at night, simultaneously with Class III elastics (Figure 4). The point of force application was the upper premolars for the Sky Hook elastics and the molars for the Class III elastics. The Sky Hook force vector was parallel to the oclusal plane, and the magnitude was 400-500 g.

**Figure 5:**
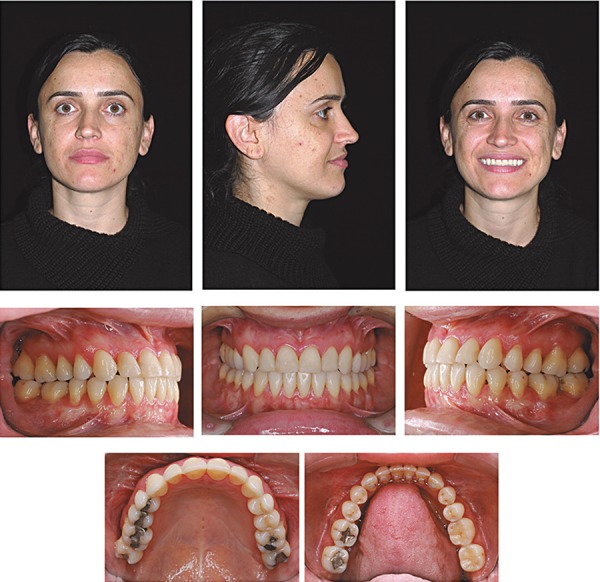
Posttreatment facial and intraoral photographs (patient signed informed consent authorizing the publication of these pictures)

**Figure 7:**
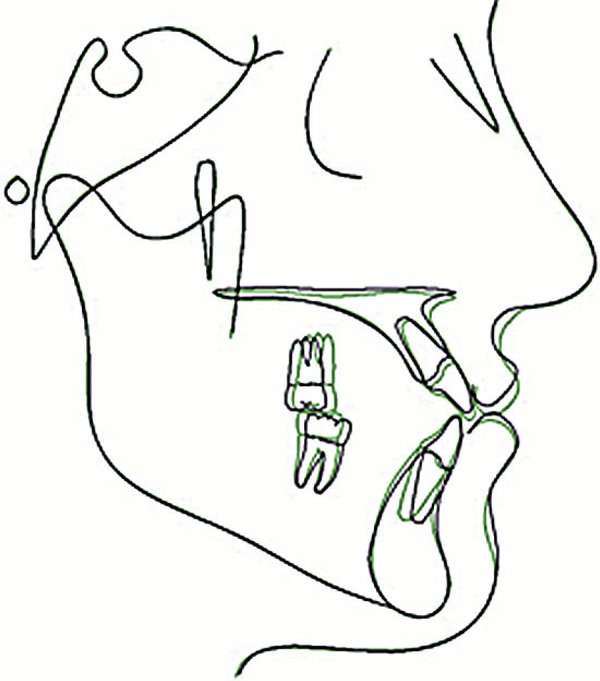
Superposition of initial and final tracings on SN at S

**Figure 8:**
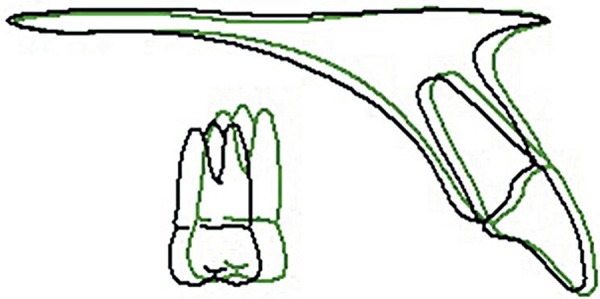
Superposition of initial and final tracings on ANS - PNS at ANS

**Figure 12:**
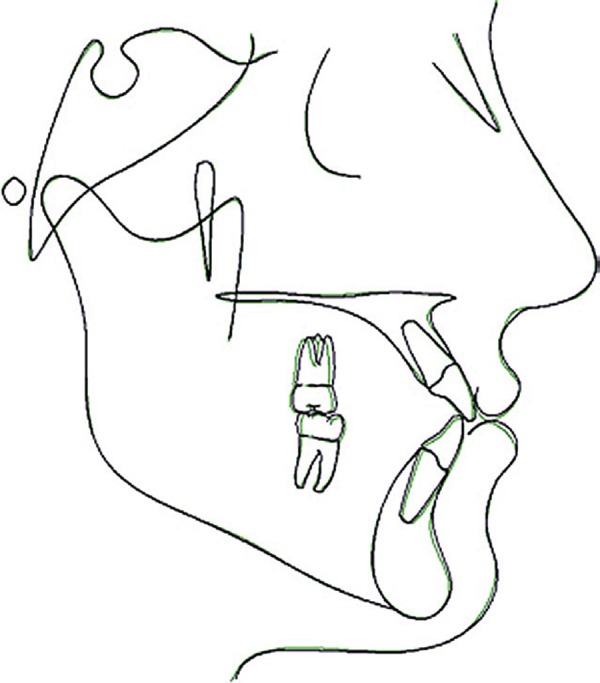
Superposition of final and follow-up tracings on SN at S

Maxillary protraction was performed during 4 month. The use of Class III elastics continued up to placement of a 0.019 X 0.025-inch stainless-steel archwire in the maxillary and mandibular arches, respectively. Patient compliance in using the elastics was excellent. After a good occlusal relationship was attained, with canine and molar Class I relationship, detailing and finishing were undertaken. Total treatment time was 33 months. On the day of debonding, a maxillary Hawley retainer was delivered, and a mandibular canine-to-canine retainer was bonded (Figure 5). She wore the Hawley retainer continuously for the first year, and only at night the next year. The lingual retainers will be kept permanently to enhance long-term stability. At the end of treatment and at 2 years and 9 months following the treatment, lateral cephalograms were traced, and changes were evaluated by superimposition of the new tracings on the pre-treatment tracings (Figures 7, 8 and 12).

**Figure 6:**
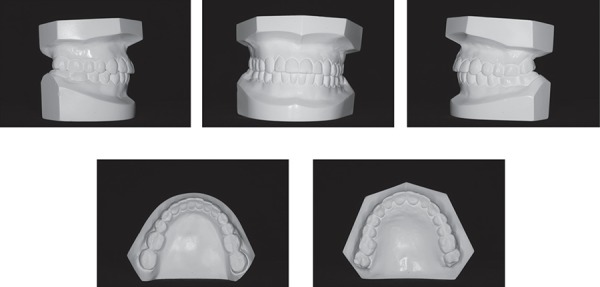
Posttreatment study models

There was improvement in the relationship between the upper and lower lips, and in the nasolabial angle, associated with projection of the middle third of the face (Figure 5). Posttreatment intraoral photographs and dental casts show satisfactory dental alignment, anteroposterior relationship, normal overjet, overbite, and transverse relationship (Figures 5 and 6). The patient was satisfied with her teeth and profile. Good intercuspation and interproximal contacts were achieved (Figures 5 to 6). The final cephalometric tracing and superimposition show that the maxillary incisors were slightly retruded and palatally tipped, the maxillary molars were mesially displaced, and the mandibular molars were distally tipped. The mandibular incisors were bucally tipped (Figures 7 and 8 and Table 1).

**Figure 9:**
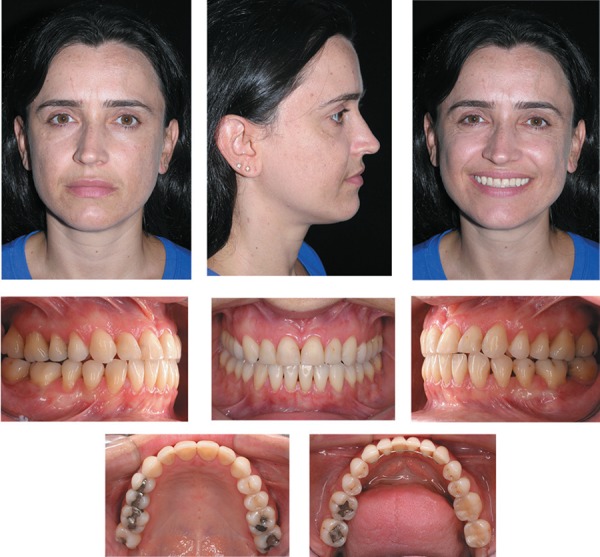
Follow-up facial and intraoral photographs (01/16/2008) (patient signed informed consent authorizing the publication of these pictures)

The follow-up results, 2 years and 9 months after the end of treatment, are shown in Figures 9-12. Facial esthetics improvement in the frontal and lateral view was maintained in the retention period. The posttreatment occlusal stability is good, with no apparent changes in the follow up. Posttreatment and follow-up superimposed tracings demonstrate slight dental and skeletal changes in the maxilla and mandible. Minimum anteroposterior changes of incisor position and maxillary protraction relapse can be observed.

## DISCUSSION

Class III malocclusion in an adult patient can be corrected without surgery, with dentoalveolar compensation^3,6-8^. However, the surgical correction provides better esthetic results and normal jaw relationship. SARMe followed by orthopedic protraction of the maxilla is an alternative able to improve the anteroposterior jaw relationship consequent to some orthopedic change.

Both Pelo, et al.^15^ (2007)and Carlini, et al.^2^ (2007) fractured the pterygomaxillary suture, which explains the greater maxillary advancement compared to our results. The SARME can be likewise performed either under general or local anesthesia, with the same procedure and the same effectiveness, except for pterygomaxillary detachment, which is absolutely unadvisable under local anesthesia, due to the possible complications and to the enormous discomfort for the patient^18^.

However, the Piezosurgery^®^ (Mectron Medical Technology, Carasco, Italy) can be an alternative for patients reluctant to undergo general anesthesia but would be beneficiated by pterygomaxillary suture separation. The Piezosurgery^®^ is selective for mineralized structures, with no effect on soft tissues. In addition to that, the separation of the pterygoid plates from the maxilla seems to be a reliable procedure if performed with the piezoelectric osteotome, because the osteotomic action of ultrasounds is very effective with this bone thickness^17^.

The proposed treatment approach was able to slightly protrude and retrude the maxilla and the mandible, respectively (SNA, SNB), improving the anteroposterior jaw relationship (ANB). However, some of these changes may have been consequent to the maxillary incisors palatal tipping and labial tipping of the mandibular incisors. The treatment was finished with a not perfect molar Class I relationship (Figures 5 and 6). However, considering the realistic targets of an adult treatment, the oclusal achievements were considered satisfactory.

**Figure 3:**
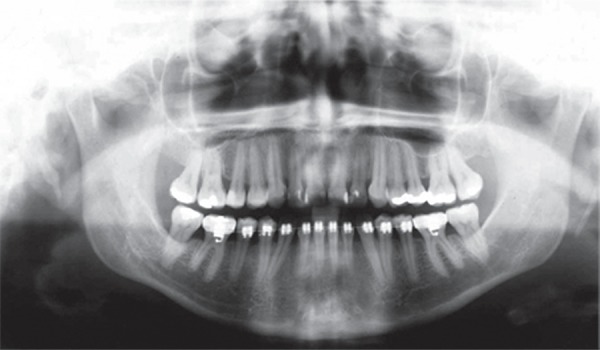
Pretreatment panoramic radiograph

**Figure 10:**
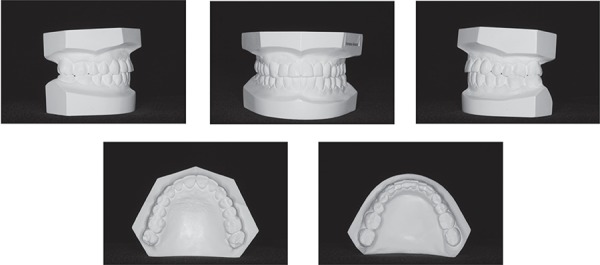
Follow-up study models

**Figure 11:**
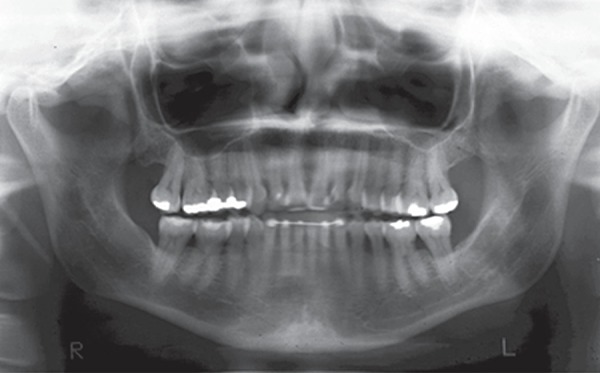
Follow-up panoramic radiograph

The dentoalveolar changes usually expected in a camouflage treatment (Class III compensation) can improve the soft tissue profile, with protrusion of the upper lip and slight retrusion of the lower lip^9,10^. Nevertheless, the patient's excessive Class III natural compensation jeopardized her appearance (Figure 1). In addition to the maxillary protrusion, the accentuated labial inclination of the maxillary incisors was corrected. The maxillary protrusion provided a satisfactory occlusal result, and the labial incisor inclination correction provided satisfactory esthetic results, with increase in the nasolabial angle. even with mandibular canine slots angulated 0° and mandibular incisors with lingual crown torque (-6°), the mandibular incisors were labially tipped. This contributed to improve the mento-labial sulcus (Figure 5). The upper and lower incisors inclined in the opposite direction for what would be expected in the Class III treatment. This result can be explained due to the excessive natural Class III compensation at the beginning of treatment, which was reduced with the pre-adjusted fixed appliance. In addition, the Sky Hook produced body movement of the incisors, which did not increase labial tipping. The clinical and radiographic follow-up examination performed 2 years and 9 months after the end of therapy confirms stability of facial esthetics improvement, which was maintained due to the stable orthopedic and oclusal outcomes (Figures 3, 9-12). Pelo, et al.^15^ (2007)also observed stable results in a 5-year follow-up of two young patients treated with a Delaire-type facemask in combination with maxillary corticotomy.

Good patient compliance was crucial for the good results achieved in the present case. This protocol is discouraged in non-compliant patients, and even compliant patients must be highly motivated.

## CONCLUSION

The choice of treatment for any malocclusion must be tailored to each patient. All treatment possibilities, including those that are ideal and those that are a compromise, should be considered and explained to the patients, so that they can choose the best possible option that offer good outcomes, while meeting their expectations and respecting their desires. In view of patient reluctance to undergo general anesthesia, SARME followed by orthopedic protraction of the maxilla can be a viable alternative in similar cases. The patient’s chief concern was addressed and treated to her satisfaction.
